# Glucocorticosteroids and the Risk of NAFLD in Inflammatory Bowel Disease

**DOI:** 10.1155/2022/4344905

**Published:** 2022-05-11

**Authors:** Sara Jarmakiewicz-Czaja, Aneta Sokal, Piotr Pardak, Rafał Filip

**Affiliations:** ^1^Institute of Health Sciences, Medical College of Rzeszow University, 35-959 Rzeszow, Poland; ^2^Institute of Medicine, Medical College of Rzeszow University, 35-959 Rzeszow, Poland; ^3^Department of Gastroenterology with IBD Unit, Clinical Hospital, No. 2, 35-301 Rzeszow, Poland

## Abstract

Each year, the incidence of nonalcoholic fatty liver (NAFLD) disease increases. NAFLD is a chronic disease. One of the most common causes of NAFLD is an inadequate lifestyle, which is characterized by a lack or low physical activity and eating highly processed foods rich in saturated fat and salt and containing low amount of fiber. Moreover, disturbances in intestinal microbiome and the use of certain drugs may predispose to NAFLD. NAFLD is an increasingly described disease in patients with inflammatory bowel disease (IBD). Recent data also indicate a frequent coexistence of metabolic syndrome in this group of patients. Certain groups of drugs also increase the risk of developing inflammation, liver fibrosis, and cirrhosis. Particularly important in the development of NAFLD are steroids, which are used in the treatment of many diseases, for example, IBD. NAFLD is one of the most frequent parenteral manifestations of the disease in IBD patients. However, there is still insufficient information on what dose and exposure time of selected types of steroids may lead to the development of NAFLD. It is necessary to conduct further research in this direction. Therefore, patients with IBD should be constantly monitored for risk factors for the development of NAFLD.

## 1. Introduction

Nonalcoholic fatty liver disease (NAFLD) is a chronic disease characterized by the presence of lipids in hepatocytes without the cause of secondary constriction and the absence of cell damage in the form of progressive fibrosis [1]. Triacylgricelore (TG) constitutes a significant part of the accumulated fat in hepatocytes [2]. A progressive form of NAFLD can lead up to 30% of NAFLD patients to develop nonalcoholic steatohepatitis (NASH) [3]. NASH can lead to organ fibrosis, which can cause hepatocellular carcinoma and cirrhosis. The primary form of NAFLD does not show any disease manifestations and is most often diagnosed during routine biochemical tests [4]. Additionally, NAFLD and NASH may increase the risk factor for the incidence of hepatocellular carcinoma and intrahepatic cholangiocarcinoma (iCCA) [5, 6].

NAFLD is an increasingly described disease in patients with IBD. IBD is a chronic condition characterized by periods of exacerbation and remission. In Crohn's disease (CD), inflammatory changes occur in sections and can be localized in any part of the gastrointestinal tract. The inflammation covers the entire thickness of the gastrointestinal wall. In colitis ulcerosa (UC), the inflammatory changes are continuous and involve the mucosa of the colon and/or rectum [7].

**Figure 1:**
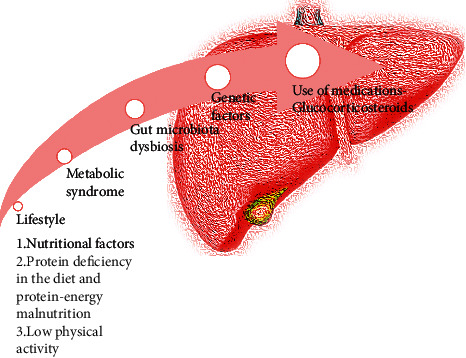
Potential risk factors affecting NAFLD development in IBD patients described in this review.

Many data confirm the influence of the presence of metabolic syndrome, obesity, or type II diabetes on the risk of NAFLD development [8]. However, the relationship between these disorders and NAFLD may be two-way [9, 10]. Previous studies have already suggested considering NASH as a part of metabolic syndrome due to the common pathophysiological mechanisms responsible for their development [11]. The most common components of the metabolic syndrome are obesity, hypertension, and carbohydrate metabolism disorders. In turn, insulin resistance is associated with the hepatic metabolism of fatty acids and the occurrence of dyslipidemia [12]. NAFLD and metabolic syndrome are increasingly described in patients with IBD [7]. In addition, NAFLD is increasingly common in younger people with IBD than in those without IBD [13] (Figure 1).

### 1.1. Epidemiology

It is estimated that 1.7 billion people worldwide are diagnosed with NAFLD [14]. The prevalence of NAFLD is higher in men than in women [15]. NAFDL is a significant clinical problem because it positively correlates with obesity [16]. Younossi et al. demonstrated that NAFLD may occur in a quarter of people in the world population [17]. Most diagnosed cases are reported in Asia, then in North America, and mainly in the United States [18]. In Europe, it is estimated that around 25% of people can develop NAFLD [19]. The incidence of the liver disease varies across Europe. The highest percentages of people with liver disease are recorded in Romania, Austria, Luxembourg, and Hungary [20]. Health care strategies should be developed and implemented to detect NAFLD in the early statistics and prevent its development in many countries [21]. The incidence of NAFLD coexistence with IBD varies. Depending on the diagnostic criteria adopted by researchers, it is estimated to occur in 40% of IBD patients [13]. However, according to Sartini et al., patients with IBD may have different NAFLD phenotypes [22].

## 2. Potential Causes of NAFLD Development in IBD

### 2.1. Lifestyle

According to the meta-analysis of data collected from 2000 to 2018 by Perdomo et al., excessive consumption of carbohydrates and simple sugars [23] with low dietary fiber supply is closely related to the occurrence of NAFLD [24, 25]. A study by Rietman et al. confirmed an inverse correlation between fiber consumption and the risk of NAFLD [26]. It may be the result of its positive effect on the reduction of postprandial glycemia and the ability to bind bile acids and their salts and then to excrete fatty acids [27, 28]. Dietary fiber is also used by intestinal bacteria to produce short-chain fatty acids (SCFA) because of which it regulates lipid metabolism in the liver and improves insulin sensitivity [27].

#### 2.1.1. Nutritional Factors

A high supply of saturated and trans fats as well as the consumption of highly processed food increase the risk of fatty liver disease [23]. A study by Kechagias et al. involving 18 adults assessed the effect of eating fast food in combination with a sedentary lifestyle for 4 weeks. As a result of this intervention, the subjects consumed twice as much energy with food, which increased their body weight on average from 67.3 kg to 74 kg (*p* < 0.001). An increase in the value of liver enzymes was also observed (ALAT from 22.1 U/l to an average of 97 U/l) [29]. The increase in the prevalence of overweight (20–40%) and obesity (15–40%) in the groups of patients with IBD may predispose them to NAFLD [30]. This may be due to a sedentary lifestyle and the consumption of too many calories in relation to the body's needs, as well as the presence of comorbidities that predispose to excess body weight [31, 32]. Hoffmann et al. in their study observed that NAFLD is more common in patients with IBD in old age and with excess body weight [33].

There is insufficient evidence that fructose intake comes together with an isocaloric diet may contribute to the accumulation of fat in the liver, but its excessive consumption may have a lipogenic effect and increase plasma triglycerides. The accumulation of intrahepatic lipids occurs when the rate of their “delivery” is faster than the time of release. A diet rich in fructose may promote the novo lipogenesis, which means that lipids can also be synthesized from carbohydrates or other precursors [34].

Choline deficiency is also an important factor in the development of NAFLD. Low dietary intake of this component is associated with a reduced outlet of low-density lipoprotein (LDL) from the liver [35]. Additionally, there is a strong relationship between the body mass index (BMI) and the degree of steatosis and choline consumption. In subjects who increased their choline intake from 272 mg/d to 356 mg/d, a reduction in the fatty liver index (FLI–calculated according to the United States FLI) was noted, and these changes were more pronounced for people with higher BMI (mean = 35.9 kg/m2.). According to Mazidi et al., the supply of choline may modulate FLI in obese people [36]. Additionally, vitamin D deficiency may also be associated with the occurrence and severity of NAFLD, but higher-quality studies are still required [37, 38].

One of the probable reasons for the high risk of NAFLD is also increased intestinal permeability, which is mainly noticed before the introduction of a gluten-free diet in patients. Liver enzymes are also elevated in the group of patients with celiac disease. In both IBD and celiac disease, there is an improper humoral reactivity against saccharomyces cerevisiae. In IBD, anti-Saccharomyces cerevisiae antibodies (ASCA) are detected well before clinical symptoms appear. Detection of ASCA in combination with perinuclear anti-neutrophil cytoplasmic antibodies (pANCA) is important serodiagnostic tests when inflammatory bowel disease is suspected. Additionally, it is believed that a positive ASCA result may be associated with the occurrence of silent celiac disease [39, 40].

On the other hand, the use of a gluten-free diet in the course of celiac disease may, to some extent, increase the risk of NAFLD. Incompetent use of this diet may be associated with worsening glucose tolerance and an increase in serum lipids. In their work, Tovoli et al. show that approximately 30% of celiac patients who follow a gluten-free diet were also diagnosed with NAFLD, which increases the risk three times compared to the general population. However, the authors point out that more research is needed in this area. In addition, it is worth carrying out a dietary consultation, which would help adjust the proper nutrition for the patient [41].

#### 2.1.2. Protein Deficiency in the Diet and Protein-Energy Malnutrition

Authors of other studies indicate underweight is a predisposing factor to impaired liver enzyme activity, which may influence the subsequent development of NAFLD in IBD patients [42]. Similar conclusions were drawn by Kang et al.; in their study, they analyzed the relationship between the occurrence of NAFLD in IBD patients and sarcopenia. They showed that loss of muscle mass might be an independent factor in the incidence of NAFLD in IBD patients. They also indicated the need for adequate nutrition in patients to prevent the worsening of sarcopenia [43] because protein and energy malnutrition is often observed in IBD patients [44]. Both malnutrition and protein deficiency may affect the development of steatosis and hepatitis [45]. A reduced supply of this component with the diet, below 9% of the total energy requirement, may lead to increased accumulation of TG in the liver and to the development of steatosis and severe inflammation. As a result of malnutrition and an insufficient amount of protein in the diet, peroxisomes and their dysfunction may be lowered, as well as mitochondrial function may deteriorate. Both hepatic peroxisomes and mitochondria are involved in the metabolism of nutrients by providing substrates for aerobic metabolism and lipogenesis by early regulation of fatty acid metabolism [46, 47]. Therefore, an adequate supply of protein and amino acids is essential for the regeneration of hepatocytes, thus preventing the accumulation of fat in the liver [45].

#### 2.1.3. Low Physical Activity

An important factor in the development of NAFLD is also low physical activity, including the lack of adapted aerobic activity [48]. According to Ahmed et al., NAFDL patients are characterized by a significantly lower rate of physical activity [49]. In an animal model study, fatty acid oxidation in the liver decreased 173 hours after the cessation of physical activity [50]. Low circulatory and respiratory efficiency significantly negatively correlates with the content of hepatic fat and increases the risk of NASH [51]. In the study by Pälve et al., participants with similar body weight but low physical activity had a higher incidence of the fatty liver compared to the control group [52]. Current data show that physical activity, both directly and indirectly, by influencing the gut microbiome, may be associated with the occurrence of NAFLD, but there are no studies that could explain this exactly [53].

### 2.2. Intestinal Microbiota

One of the frequently observed pathological changes in IBD patients is intestinal dysbiosis, that is, a decrease in commensal microbiota, and an increase in the amount of pathogenic microorganisms. In patients, exposure to unfavorable environmental factors, for example, selected food additives, may reduce the mucus layer and break the tight junction because of which pathogens enter the body. The body's immune cells (T cells, macrophages, and dendritic cells) enter the site of the entry of microorganisms, and there is a “release” of proinflammatory cytokines, which predisposes to the development of inflammation [54]. A significant proportion of nutrients, bacteria, and metabolites pass from the intestines along with the blood through the portal vein to the liver [35].

As a result of the activation of Kupffer cells and inflammatory cytokines by nonpathogenic strains of *Escherichia coli*, as well as proteolytic bacteria (mucus and lactose negative strains of *E. coli*, *Clostridium* spp., and *Serratia* spp.), nitrogen compounds are metabolized, which are then transformed into indole, ammonia, and skatole and may be cytotoxic to liver cells. Moreover, the presence of fungi, mainly *Candida albicans*, may lead to the development of hepatosplenic candidiasis [35, 55]. Patients with NAFLD also have increased intestinal permeability, which may lead to the migration of bacteria, which results in an increase in LPS levels [56]. LPS may contribute not only to the development of metabolic endotoxemia and the weakening of the intestinal barrier [57] but also to the development of obesity, insulin resistance, and diabetes [58]. Similarly, Miele et al. showed that in the NAFLD breakthrough, there is also increased intestinal permeability and dysbiosis. The authors also indicated that it may be related to disturbances in lipid metabolism [59]. According to Wisnewsky et al., LPS can cause inflammation by producing, for example, trimethylamine N-oxide, which may predispose to NAFLD [60]. Chen et al. also pointed to the association of dysbiosis with the development of NAFLD by reducing *Bacteroidetes* and increasing *Proteobacteria* [61] as in the course of IBD [62, 63]. In an animal model, the use of a diet high in sucrose and fat was associated with an increased level of circulating LPS, IL-6, and TNF-*α* and an increase in alkaline phosphatase in intestinal tissue homogenates [64]. The excessive supply of fructose may also contribute to endotoxemia and increased lipid accumulation in the liver, bacterial overgrowth, and increased intestinal permeability [65–67].

Likhitsup et al. in their study presented the prevalence of NAFLD with coexisting IBD and NAFLD itself. People who abused alcohol and used steroids were excluded from the study. The authors indicated that patients with IBD were diagnosed with NAFLD much more often (44%) compared to the group without IBD (16%). In this case, NAFLD may be caused by dysbiosis of the intestinal microbiome and an active disease process in the body [68]. Liu et al. in their work show that SCFA have a beneficial effect on IBD and NAFLD due to the promotion of reconstruction and strengthening of the intestinal barrier [69]. Many authors point to the severity of disease activity and a longer duration of IBD as predictors of NAFLD occurrence [70, 71].

### 2.3. Metabolic Syndrome (MS)

The metabolic syndrome, also known as syndrome *X*, is a group of clinical conditions that include abdominal obesity, systemic hypertension, insulin resistance, and atherogenic dyslipidemia [72]. Yorulmaz et al. examined 177 patients with IBD, 62 with CD, and 115 with UC. The metabolic syndrome was confirmed in 12 of 117 (10.3%) patients with IBD aged less than 45 years and in 33 of 60 (55%) patients aged 45 years and over. In addition, the concentration of C-reactive protein and the concentration of uric acid were significantly higher in patients with UC and insulin resistance [73]. C-reactive protein is a marker of inflammation, and it is produced in the liver by the secretion of the cytokines IL-6, IL-1, and TNF-*α* [12]. In the study by Fröhlich et al., increased levels of CRP and IL-6 were observed in the course of MS [74]. Elevated levels of CRP and IL-6 closely correlate with the development of fatty liver [75]. Based on this, it can be assumed that IBD patients who develop metabolic syndrome are also at risk of developing fatty liver disease.

The risk of developing fatty liver intensifies with the increase in the BMI value and the occurrence of central obesity. Disturbances in lipid metabolism are also observed in this group of patients. In people with NAFLD and/or MS, the rate of VLDL lipoprotein secretion is much higher than in people without these disorders. In turn, the impaired ability of insulin to inhibit glucose production in MS patients leads to the occurrence of hyperglycemia and increased insulin secretion, which may lead to the development of type II diabetes, which may predispose to the progression of NAFLD [9].

According to the data collected by Younossi et al., the incidence of NAFLD in the group of 49,419 patients with type II diabetes was 55.5% [76]. On the other hand, the presence of NAFLD may be a factor indicating an increased risk of T2DM, regardless of age or degree of obesity. Moreover, insulin resistance of liver cells plays an important role in the pathogenesis of the disease [77], which is associated with increased lipid synthesis in the liver, thus leading to the development of hypertriglyceridemia and hyperglycemia [78].

A 2018 meta-analysis of studies found that NAFLD doubles the risk of developing diabetes. However, there are still no studies that would confirm the causal relationship [79]. The intrahepatic and extrahepatic pathways, mediated by insulin, play a significant role in controlling lipid and glucose metabolism. There is an increased production of glucose in the liver due to increased lipogenesis in the course of hepatic insulin resistance. The hepatic action of insulin on the liver is believed to be mediated via the phosphoinositol-3-phosphate kinase (PI3K/Akt) signaling pathway, which mediates the effects of insulin on anabolic metabolism. Activation of Akt by insulin may inhibit glycogenolysis and gluconeogenesis, inter alia, by glycogen synthase kinase 3 enzyme (GSK3) and forkhead box protein O1 (FoxO1) [78].

### 2.4. Hepatitis C

According to research, hepatitis C is closely related to the development of NAFLD. Infection with HCV, especially with genotype 3, may lead to disorders of lipid metabolism in hepatocytes, and the severity of steatosis depends on the replication capacity of the virus. Additionally, HCV is positively associated with insulin resistance and the development of T2DM [80]. Nevertheless, regardless of insulin resistance, steatosis as a result of HCV infection may influence the development of oxidative stress, the severity of fibrosis, and the occurrence of hepatocellular carcinoma [81]. HCV inhibits the production and activity of microsomal triglyceride transfer protein (MTP), leading to a reduction in VLDL export and thus to the accumulation of lipids in hepatocytes. HCV may also interfere with the transcription of the alpha receptor responsible for the activation of PPAR-*α*, which induces fatty acid uptake. An important role is assigned to the protein binding sterol regulatory elements that can be activated by HCV, leading to the synthesis of fatty acids in liver cells [82].

### 2.5. Genetic Factors

The authors indicate that genetic factors pose a significant risk in the development of NAFLD. Family members of NAFLD patients in the first stage are more likely to develop the disease than the general population [83, 84]. Additionally, the presence of NAFLD is associated with some genes, including *PNPLA3*, *MBOAT7*, or *GCKR* [9, 85].

### 2.6. Use of Medications

The applied treatment may cause NAFLD in patients with IBD [86]. Certain medications can increase the risk of both obesity and NAFDL contributing to the development of inflammation, liver fibrosis, and cirrhosis. Their hepatoxicity has been demonstrated in clinical and experimental studies. Among them, there are androgenic steroids, benzbromarone, corticosteroids, irinotecan, methotrexate, and tamoxifen [87].

## 3. Influence of Steroid Use on the Development of NAFLD

**Figure 2:**
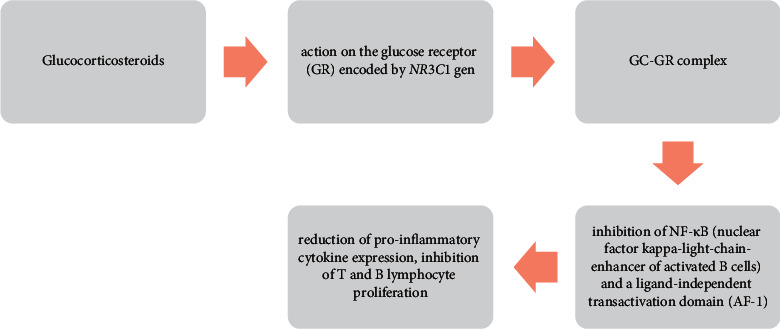
Proper operation of glucocorticosteroids as exemplified by the amino region of the GR*α* isoform.

Corticosteroids, such as corticosterone, dexamethasone, prednisone, or cortisone, are used in many diseases due to their anti-allergic and anti-inflammatory effects (Figure 2) [83]. They can be used, inter alia, in the treatment of HELLP syndrome (hemolysis, elevated liver enzymes, and low platelet count) in the antenatal period [88], in the treatment of dermatoses [89], and in alleviating pain symptoms during radiotherapy in patients with bone metastases [88]. They are also used in musculoskeletal diseases [90, 91], lupus [92], and ulcerative colitis or Crohn's disease [93, 94].

**Figure 3:**
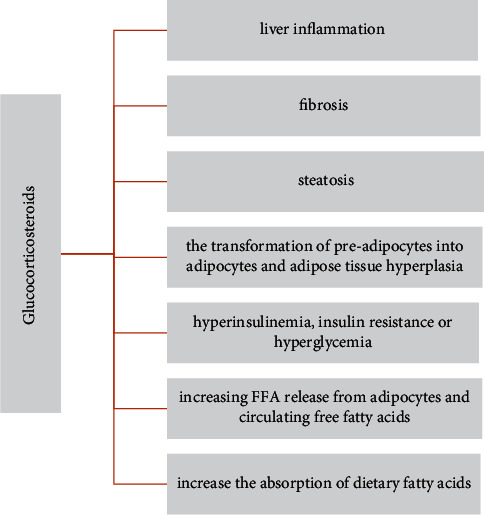
The potential side effects of using glucocorticosteroids.

Nevertheless, steroid therapy is also often associated with the occurrence of many complications, including metabolic and/or endocrine disorders such as obesity, type II diabetes, and hyperlipidaemia (Figure 3). Moreover, there are data confirming the influence of corticosteroids on the development of NAFLD, inflammation, and the occurrence of benign liver tumors [87].

### 3.1. Use of Glucocorticosteroids in IBD and the Risk of Developing NAFLD

Principi et al. concluded that NAFLD may develop in IBD patients who received early and long-term old therapy [13]. On the other hand, in the study by Sarola et al., a similar relationship has not been demonstrated; however, the authors emphasize that attention should be paid to the treatment used, as numerous studies have confirmed that the use of steroids in IBD may predispose patients to NAFLD [95]. Carr et al. also did not confirm this relationship. They showed that the presence of MS is mainly associated with the occurrence of fibrosis in NAFLD in IBD patients. The researchers found no correlation between drug use and the severity of the disease and the occurrence of fibrosis in NAFLD [96]. Lapumnuaypo et al. analyzed 7 observational studies with 1,610 patients and found no significant association between IBD treatment and the development of NAFLD in patients [97]. However, due to the complexity of the mechanism of steroid therapy on the development of NAFLD, more research is needed in this area, and current studies on the effects of steroid use in the treatment of IBD are conflicting.

### 3.2. Potential Influence of Glucocorticosteroids on the Development of NAFLD

#### 3.2.1. NAFLD Disease Phenotypes and GCs in Patients with IBD

Patients with severe IBD are more likely to develop NAFLD, which in turn may negatively affect the course of IBD [17]. The study by Mohannadi et al. involved 913 patients with CD (41.9%) and UC (58.1%). Fatty liver disease was diagnosed in 22.2% of IBD patients. Patients diagnosed with NAFLD were older, had a higher BMI, and were more likely to suffer from other diseases such as diabetes and hypertension. The biological treatment has not been shown to influence the risk of bruising [98]. Similar results were observed in the study by Sourianarayanane et al. NAFLD was detected in 8.2% of the examined people with IBD. The subjects with steatosis were older than the control group and 29% of the subjects were also diagnosed with metabolic syndrome. In addition, steroid use was an independent factor in the prevalence of NAFLD [86]. In a retrospective cohort study by Carr et al., 23% of IBD patients were also diagnosed with MS. The incidence of IBD and MS was also related to the age of the examined people. Additionally, patients with MS had higher serum levels of alanine aminotransferase (ALT) and aspartate aminotransferase (AST) and a higher value of NFS (NAFLD fibrosis score). The impact of NAFLD severity on the course of IBD has not been reported. There was also no difference between patients who used steroids and those who did not [96].

Although study results are still inconclusive, corticosteroids should be used with extreme caution in patients with risk factors of metabolic syndrome.

#### 3.2.2. GC-GR Signaling

Glucocorticosteroids (GCs) play a key role in the pathogenesis of NAFLD by directly affecting the liver and adipose tissue [99]. GCs favor the transformation of preadipocytes into adipocytes and are involved in adipose tissue hyperplasia [100]. The analysis of studies carried out by Rahimi et al. showed that excessive intake of glucocorticosteroids may lead to steatosis and other metabolic disorders, such as hyperinsulinemia, insulin resistance, or hyperglycemia [101]. It is believed that both high GCs exposure and antagonism of the glucocorticoid receptor (GR), a member of the nuclear hormone receptor family, may promote the development of steatosis [102, 103]. GC-GR signaling is important in the maintenance of blood glucose, and GCs can impair its uptake in skeletal muscle and adipose tissue. Due to their opposite effect on insulin, elevated GCs levels may lead to insulin resistance [104, 105]. On the other hand, treatment of GCs may resemble Cushing's disease, which is associated with elevated cortisol levels and often leads to insulin resistance not only in peripheral tissues but also in the liver. Therefore, patients with Cushing's syndrome are characterized by metabolic syndrome [106].

**Figure 4:**
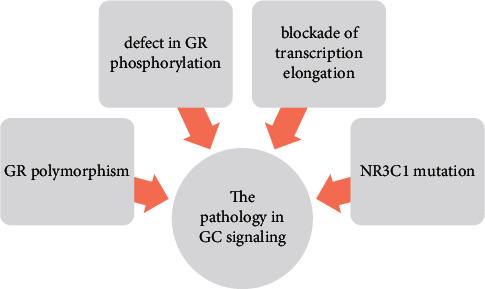
The causes of the pathology in GC signaling.

The cause of the pathology in GC signaling (Figure 4) may be the NR3C1 mutation, which predisposes to the development of metabolic disorders. GR polymorphisms may also be associated with GC resistance. The GR mutation can lead, inter alia, to a defect in GR phosphorylation or blockade of transcription elongation; therefore, GC signaling may be disturbed at any stage of molecular activity [107]. Aranda et al. concluded that GR located in the intestinal epithelium plays a significant role in maintaining normal colon homeostasis. The deletion of GR NR3C1 can alter the intestinal barrier function and induce local inflammation, which may “pass/spread” to systemic inflammation [108].

Previous studies have shown no effect of corticosteroids on liver function and no changes in FFA, bilirubin, and beta–OH–butyrate levels or in enzyme activity [109, 110]. However, it has been found that GCs can increase free fatty acids (FFA) released from adipocytes and increase the circulating of FFA with elevated insulin levels, while their moderate concentration leads to inhibition of lipogenesis in adipocytes [111, 112]. Feng et al. in their study on animal models showed that long-term administration of corticosterone (CORT) influences the induction of liver inflammation and fibrosis. The authors point to the role of m6A (N6-methyladenine) in the post-transcriptional regulation of heat shock proteins (HSPs), which are induced in response to severe stress [113].

GCs exert a strong influence on energy homeostasis and the regulation of metabolic pathways in the liver, including FFA uptake, de novo lipogenesis, VLDL lipid export, and inhibition of *β*-oxidation of fatty acids [102]. Accumulation of TG in the liver may also occur as a result of a decrease in the degradation of apolipoprotein B and reduced activity of triacylglycerol hydrolase, an enzyme that hydrolyses TG in hepatocytes [114, 115]. In addition, GCs can inhibit the transcriptional activity of PPAR-*α* and thus reduce *β*-fatty acid oxidation and, consequently, increase lipid accumulation [116].

In an animal model study, Theisem et al. showed that GCs can increase the absorption of dietary fatty acids, depending on the amount and type of fatty acids supplied. Changes in lipid absorption have been observed during 4 weeks of treatment with prednisone (PRED) and budesonide (BUD) or using a diet based on saturated fatty acids (SFA) or polyunsaturated fatty acids (PUFA). Rats fed SFA showed increased lipid uptake compared to rats based on PUFA diets [117]. In another study, the same authors showed that BUD increased ileal linoleic acid and jejunal oleic acid uptake, and PRED increased the uptake of cholesterol, linoleic, linolenic, lauric, and palmitic acid. Moreover, they concluded that fatty acid uptake occurs irrespective of the early response of genes or genes encoding cytokines, proglucagon, and fatty-acid-binding protein (FABP) [118].

However, there is still no data on what dose and duration of exposure to GC may lead to the development of steatosis, especially in humans. The effect of GR-GR signaling is not well understood and described, and the available studies have been conducted mainly in animal models.

## 4. NAFLD Treatment

Due to the fact that obesity is one of the main causes of NAFLD, treatment of patients with excess body weight should focus on introducing a healthy lifestyle [119]. In numerous studies, the authors show that initially, eating habits should be changed and physical activity should be introduced in order to reduce body weight. If these methods fail, they suggest that the next step is the pharmacological treatment of obesity; otherwise, the patient may only be referred for bariatric treatment [120]. A 3–5% reduction in body weight improves the parameters of NAFLD by reducing organ steatosis, while a 7–10% reduction in body weight has also shown a beneficial effect on NASH [3]. Polyzos et al. in their work describe that a beneficial effect may reduce the caloric content of the diet by 500–1,000 kcal per day. In addition, it is advisable to avoid foods that may increase the risk of obesity, thus worsening NAFLD (highly processed foods, sugar, sweets, sweet drinks, salty snacks, products containing significant amounts of saturated fat, etc.) [121]. Physical activity should be individualized for each patient, adjusted to their preferences, which will contribute to long-term lifestyle changes. Physical exercise has shown beneficial effects on NAFLD through, inter alia, improvement of fatty acid metabolism or reduction of insulin resistance [122–124].

### 4.1. Pharmacological Treatment

Pharmacological treatment of NAFLD affects, in particular, the improvement of insulin resistance, glycemia, and fat metabolism by reducing obesity. Ranjbar et al. indicated the beneficial effect of GLP-1 RAs (glucagon-like peptide-1 analogs), SGLT2i (sodium-glucose cotransporter 2 inhibitors), and DPP-4i (inhibitors of dipeptidyl peptidase 4) on biochemical and histopathological parameters of NAFLD and NASH [125]. Authors of other studies also point to the therapeutic properties of GLP-1 RA. These are drugs used in type II diabetes. Liraglutide, which belongs to the group of GLP-1 analogs, and sitagliptin, which belongs to DPP-4 inhibitors, have beneficial effects on the liver [104, 126]. Singh et al. in their review also presented potential therapeutic options that could be included in the treatment of NASH in the future. The authors list here, inter alia, ASK1 inhibitors, p38 MAPK inhibitors, PPAR-*α*, and PPAR-*δ* agonists [127].

### 4.2. Surgical Treatment

Weight loss due to bariatric treatment may reduce steatosis, hepatitis status, and organ fibrosis [128]. In addition, the ALT and AST profiles can be improved [129]. However, bariatric surgery is not indicated for all NAFLD patients. People with morbid obesity should be referred for the procedure if the first and second steps did not meet the expectations [130]. Due to the complexity and varying degrees of gastrointestinal intervention, the most effective type of bariatric treatment for NAFLD patients has not been established yet. On the other hand, the authors emphasize the effectiveness of bariatric surgeries in this group of patients [131].

### 4.3. Probiotic Therapy

Another way to treat NAFLD is to normalize/restore a beneficial gut microbiome. Changing the intestinal microbiota is primarily aimed at normalizing lipid metabolism.

Ritze et al. showed that in animal models, LGG increases the amount of commensal bacteria, restores the proper function of tight junction proteins, and may reduce the amount of proinflammatory cytokines (e.g., IL-1*β*) [132]. Famouri et al. applied probiotic therapy with *Lactobacillus acidophilus*, *Lactobacillus rhamnosus*, *Bifidobacterium bifidum*, and *Bifidobacterium lactis* strains for 12 weeks in children with NAFLD. After the intervention, researchers found a decrease in the level of hepatic enzymes (ALT and AST) as well as in the level of total cholesterol and TG [133]. Liu et al. in their meta-analysis indicate that both probiotics and synbiotics (probiotics and prebiotics) show a beneficial effect in lowering liver enzymes and improving the lipid profile in NAFLD patients [134]. Sharpton et al. concluded that the use of probiotics and synbiotics was associated with a reduction in the markers of hepatitis and its steatosis in people with NAFLD [135]. In a 12-week study by Ruscica et al., one group was given Bifidobacterium longum with nutraceuticals and the other group got placebo. They noticed that in the first group, the concentration of total cholesterol and LDL cholesterol was lowered, which may indicate a decrease in the synthesis (without greater absorption) of cholesterol [136]. Loman et al., in their meta-analysis, looked for a relationship between pre- and probiotic treatment and NAFLD. They observed a decrease in BMI; liver enzymes such as ALT, AST, and serum total; and LDL cholesterol. The authors also suggest that pro- and prebiotic therapy may be a potential treatment for NAFLD patients. However, they also point out that more research is needed in this area [137]. Chen et al. presented the possibility of modulation of the intestinal microflora and its metabolites using probiotics containing strains such as *Lactobacillus casei*, *Lactobacillus plantarum*, and *L. rhamnosus* GG [61]. Other studies indicate strains such as *L. acidophilus* L1, *Lactobacillus gasseri* BNR17, *Bifidobacterium breve*, *Bifidobacterium infantis*, and *B. longum*, which have beneficial effects in the treatment of NAFLD. The preferred prebiotics are inulin and fructo-oligosaccharides [138–140]. Due to the potential cause of the development of NAFLD and IBD, that is, dysbiosis, supportive therapy with the use of probiotics/and prebiotics should be considered.

### 4.4. Vitamin E

The therapeutic effect of vitamin E is also indicated as a factor that can improve the progressive form of NAFLD, that is, NASH. Vitamin E has an anti-oxidant, anti-apoptotic, and anti-inflammatory function. In the results of their meta-analysis, Ji et al. observed a decrease in the values of ASP and ALT in NAFLD patients after vitamin E supplementation [141]. Vitamin E, due to its anti-oxidant properties, significantly reduces oxidative stress, which is often mentioned by researchers as a factor that may predispose to the progression of inflammation in NASH [142]. In addition, anti-oxidant levels in existing liver disease can be significantly lowered due to the increased amounts of free radicals that result from excess fatty acids in hepatocytes and the malfunctioning of mitochondria [143, 144].

### 4.5. Physical Activity

Physical activity may promote weight loss and positively affect the reduction of adipose tissue, including visceral tissue, and intraliver fat, contributing to the reduction of cell resistance to insulin [145, 146]. Increased physical activity may also reduce the risk of cardiovascular diseases and disorders of the lipid profile [147, 148]. Physical activity increases fatty acid oxidation and reduces the synthesis of fatty acids in the liver, thus preventing mitochondrial and hepatocellular damage [124]. Zelber-Sagi et al. showed that the implementation of resistance training at least once a week may help reduce the abdominal obesity index [149]. In turn, according to Sreenivas Bab et al., moderate aerobic exercise may influence the normalization of ALT levels in patients with NASH [150]. For this reason, regular physical activity in NAFLD patients seems necessary to support the treatment process.

## 5. Conclusion

There are many risk factors for developing nonalcoholic fatty liver disease in patients with IBD. Both excess body weight and malnutrition, especially protein and energy malnutrition, can contribute to the development of fatty liver and hepatitis. In addition, improper eating habits and low physical activity contribute to the intensification of fat accumulation in the liver cells. Thus, a positive energy balance in the diet and consumption of highly processed food may indirectly lead to disturbances of the intestinal microbiota, which also poses a potential risk of NAFLD development.

It is assumed that long-term intensive steroid therapy may lead to NAFLD. However, there is still a lack of data on what dose and duration of exposure of selected types of steroids may lead to the development of NAFLD in humans. Due to the use of this group of drugs, steroids are also indicated as a possible cause of NAFLD development in IBD patients, but further research in this direction is necessary.

Appropriate probiotic therapy should be considered in patients with NAFDL, especially in the course of IBD. The introduction of such therapy may have a positive effect on the reduction of liver enzymes and improvement of the lipid profile in this group of patients.

It seems justified to constantly monitor patients in terms of risk factors for the development of NAFLD and to introduce appropriate nutritional management and physical activity as part of the prophylaxis of the development of the fatty liver.
